# Avoidant Personality Traits and Avoidant Coping in Cognitive–Behavioral Therapy vs. Short‐Term Psychodynamic Psychotherapy for Adult Depression

**DOI:** 10.1002/pmh.70081

**Published:** 2026-05-03

**Authors:** Saskia de Bruin, Pauline L. Herrmann, Jack J. M. Dekker, Jaap Peen, Henricus L. Van, Frank Don, Ellen Driessen

**Affiliations:** ^1^ Pro Persona Mental Health Care Nijmegen the Netherlands; ^2^ Department of Clinical Psychology, Psychotherapy and Psychoanalysis, Institute for Psychology Klagenfurt University Klagenfurt Austria; ^3^ Faculty of Behavioural and Movement Sciences Vrije Universiteit Amsterdam Amsterdam the Netherlands; ^4^ Arkin Mental Health Care Amsterdam the Netherlands; ^5^ Depression Expertise Center Pro Persona Mental Health Care Nijmegen the Netherlands; ^6^ Department of Clinical Psychology, Behavioural Science Institute Radboud University Nijmegen the Netherlands

**Keywords:** avoidant coping, avoidant personality, cognitive–behavioral therapy, depression, dimensional assessment, short‐term psychodynamic psychotherapy

## Abstract

Research on the significance of comorbid personality disorders (PD) on the outcome of depression treatment has shown inconsistent findings. In addition, it is still unclear whether treatment choice based on personality traits and coping can enhance the efficacy of depression treatment. Aiming to deliver clinically representative results, we use dimensional measures to examine avoidant personality and coping as moderators for the efficacy of cognitive–behavioral therapy (CBT) versus short‐term psychodynamic psychotherapy (STPP) for depression. Furthermore, we explored whether these depression treatments reduced avoidant personality traits and coping. Included were 265 patients with major depressive disorder who received 16‐week CBT or STPP in a randomized clinical trial. Depression, avoidant personality traits, and avoidant coping were measured with, respectively, the Hamilton Depression Rating Scale, NEO Five Factor Inventory (extraversion and neuroticism subscales), and Utrecht Coping List (avoidance subscale). Multilevel regression analyses estimated the moderating effects of avoidant personality traits and avoidant coping on the relationship between treatment type and depressive symptom change, as well as changes in avoidant personality traits and avoidant coping in CBT and STPP. Avoidant personality traits and avoidant coping did not moderate the efficacy of CBT and STPP. Both treatments resulted in significant reductions in avoidant personality traits, but not in coping. Both CBT and STPP can be offered to patients with avoidant personality traits and avoidant coping and can reduce avoidant personality traits.

**Trial Registration:** ISRCTN31263312 (http://www.controlled‐trials.com).

## 1 Introduction

Almost half of the patients suffering from depression also meet DSM‐5 criteria for personality disorders (PD) (American Psychological Association, APA 2019). Being diagnosed with depression and comorbid personality pathology is associated with lower levels of daily functioning, increased recurrence, and more rapid relapse rates, as well as higher nonresponse to treatment compared with depression only (Mulder et al. 2022; Newton‐Howes et al. 2006; Newton‐Howes et al. 2014). Given the enormous impact of depression and personality pathology on patients and families, as well as considerable socioeconomic and healthcare costs (König et al. 2020; Christensen et al. 2020; Sveen et al. 2023), quick access to suitable (cost) effective treatment is highly relevant.

Guidelines describe several effective treatments for major depression, with a choice between cognitive–behavioral therapy (CBT), interpersonal therapy (IPT), short‐term psychodynamic psychotherapy (STPP), behavioral activation, or antidepressant medication (American Psychological Association, APA 2019; World Health Organization, WHO 2020; National Institute for Health and Care Excellence, NICE 2022). Approximately 40% of depressed patients included in randomized controlled trials with follow‐up assessments experience a relapse after more than 2 years, with patients treated with psychotherapy having a higher chance of remission without relapse than those included in control conditions (Steinert et al. 2014). The most recent APA guidelines state that no specific depression treatment can be seen as more effective than another (American Psychological Association, APA 2019, ES14).

In clinical samples, where comorbidity of depression and personality pathology is common, treatment choice becomes more complex, and studies assessing the influence of personality on depression treatment show conflicting outcomes. Focusing on the treatment of patients with comorbid depression and PD, a review by Mulder (2002) concludes that in well‐designed studies, personality pathology does not alter the efficacy of depression treatment. More recently, Banyard et al. (2021) came to similar conclusions in their meta‐analysis on the efficacy of CBT depression treatment for patients with and without comorbid PD. In contrast, other studies found that PD (Danayan et al. 2024; Newton‐Howes et al. 2014; Newton‐Howes et al. 2006) or personality features like harm avoidance (Balestri et al. 2019; Mulder et al. 2022) were negatively associated with the outcome of depression treatment.

Although Mulder et al. (2022) argue that it is most effective to let treatment choice be guided by patient preference regardless of personality pathology, van Bronswijk et al. (2021) conclude that statistical prediction based on demographics or depression history is superior to clinical judgment in choosing the most effective therapeutic approach for depression. Conflicting findings about the most effective treatment for depression with comorbid personality pathology leave us with uncertainty about which treatment should be offered to the individual patient. Little is known about what differentiates patients who do and do not benefit from a certain treatment.

Focusing on this particular question, personalized medicine has become more popular (Simon and Perlis 2010)—referring to the idea that certain patient characteristics can help to select and tailor treatment that is most effective for a specific patient and is most likely to improve the individual's long‐term well‐being. Several research groups have focused on the idea of tailored treatment selection based on individual patient characteristics, with promising results: A meta‐analysis by Beutler et al. (2018) focused on fitting different types of psychotherapy to individuals' coping style, concluding that patients with an internalizing coping style benefit most from insight‐focused therapy, whereas externalizing patients should ideally be treated with symptom‐focused interventions.

Evidence is conflicting regarding differential treatment effects between CBT and other treatments in patients with either avoidant personality traits or PD from cluster C, comprising avoidant, dependent, and obsessive‐compulsive PD. Some studies found CBT to be superior to IPT (Joyce et al. 2007; Barber and Muenz 1996; Hardy et al. 1995). McBride et al. (2006) also found CBT to be superior to IPT for depressed patients with anxious and avoidant attachment. Concerning depression with comorbid anxiety disorders and anxiety symptoms, which is often characterized by avoidant coping, van Bronswijk et al. (2018) found CBT more beneficial than IPT, although this effect was no longer present in the follow‐up phase. Kikkert et al. (2016) found no treatment to be superior to the other when comparing CBT with STPP for depressed patients with avoidant PD (including subthreshold PD). All in all, studies seem to either show no difference or superiority of CBT over other psychotherapies in treating depression in patients with cluster C personality.

When analyzing personality traits for personalized treatment, dimensional rather than categorical assessments may be considered. The use of dimensional personality constructs has several advantages for clinical application as well as scientific research. Critics of the DSM‐5, which is still predominantly focused on categorical diagnoses, emphasize that PD is not a binary concept but should be viewed as a dimensional construct in clinical practice as well as research, because of (a) more statistical power in explaining study outcomes, (b) advantages in addressing issues of comorbidity and heterogeneity, and (c) the opportunity to create a comprehensive picture of an individual (Hopwood et al. 2018; Joyce et al. 2007). Researchers and clinicians are promoting the need to focus on personality concepts that are easy to adopt by investigating coping styles and personality traits with well‐known instruments like the NEO Five Factor Inventory (NEO‐FFI) and Minnesota Multiphasic Personality Inventory (MMPI‐2) instead (Newton‐Howes et al. 2006). The use of dimensional measures in personality research provides an opportunity to include patients below the current DSM threshold; i.e., many patients in clinical practice receive an unspecified or other specified (formerly “NOS”) PD diagnosis as their symptoms do not fully match one particular PD (Crawford et al. 2011; Samuel and Widiger 2008), and as a result, these individuals are often excluded from studies about matched care and treatment assignment. On the other hand, dimensional measures such as the NEO‐FFI might insufficiently differentiate between pathologically relevant traits, as they are designed to cover the range of personality traits in a broader nonclinical population. As van Bronswijk et al. (2020) concluded in their most recent meta‐analysis, there is a lack of studies assessing personality dimensionally. Currently, the field is in the midst of a paradigm shift, moving from a categorical to a dimensional approach of PD (Tyrer et al. 2025). The newly introduced 11th revision of the International Classification of Diseases (World Health Organization 2022) adopts this by no longer classifying PD by categories but by severity levels, with dimensional personality patterns added. This approach allows for gathering information about personality traits and coping at the individual patient level rather than overlapping diagnoses, which, especially in clinical practice, often remain unclear. Personality dimensions such as avoidance, being an essential concept in different psychotherapy frameworks, will persist most likely as transdiagnostic criteria for indication purposes and treatment choice. Hence, we emphasize the importance of proceeding with tailored‐treatment research using dimensional measures for concepts of personality and coping.

## 2 Objective

The current study is using dimensional measures (i.e., questionnaires identifying avoidant personality traits and coping style) instead of a categorical (i.e., DSM‐5) approach to classify avoidant personality. The first aim of this study is to examine baseline avoidant personality and coping as moderators for the efficacy of CBT versus STPP for depression. We expect to replicate previous studies' (Joyce et al. 2007; Barber and Muenz 1996; Hardy et al. 1995) finding that depressed patients with avoidant personality traits or avoidant coping style benefit more from CBT than from other psychotherapies.

Given the fact that personality symptoms and depression are closely related and reciprocally affecting one another, we furthermore aim to explore whether avoidant personality traits can be reduced by standard depression treatment, offering an opportunity to optimize treatment outcomes (Van and Kool 2020). Therefore, the second aim of this study is to investigate whether CBT and STPP as depression treatments also reduce avoidant personality traits and coping as in Emmelkamp et al. (2006) and Svartberg et al. (2004). We hypothesize that both therapies result in a reduction of avoidant personality traits and avoidant coping, but that this reduction is greater in CBT than STPP.

## 3 Method

### 3.1 Design

The current study is based on data from a randomized clinical trial (RCT) comparing CBT and STPP for adult depression. The protocol for this study was published (Driessen et al. 2007) and registered at http://www.controlled‐trials.com (identifier ISRCTN31263312). Main trial outcomes have been described elsewhere (Driessen et al. 2013, 2015, 2017). No significant differences were found between treatment conditions on any of the outcome measures, and noninferiority of STPP to CBT was shown for posttreatment measures of depression, anxiety, pain, and quality of life, as well as for anxiety measures at follow‐up. The measures of coping style and personality used for the current study were part of the routine outcome monitoring (ROM) in the clinics where the study was conducted and have not been previously reported on.

### 3.2 Participants

A total of 341 adults recruited from the patient populations of three specialized outpatient mental health clinics in Amsterdam, the Netherlands, were included in the RCT (Driessen et al. 2013). Study inclusion criteria comprised a major depressive episode (according to DSM‐IV criteria assessed with Mini‐International Neuropsychiatric Interview—Plus (MINI‐P)), a Hamilton Depression Rating Scale (HAM‐D) score ≥ 14, age 18–65 years, and written informed consent after full explanation of the study procedures. Psychotic symptoms, bipolarity, severe suicidal ideation, substance use, or medication impacting mental functions were exclusion criteria.

### 3.3 Instruments

#### 3.3.1 HAM‐D

The observer‐rated HAM‐D (Hamilton 1960) was used to assess the severity of depressive symptoms as the primary outcome variable for the moderation questions in the current study. The HAM‐D consists of 17 questions, being rated on a 0–2 or 0–4 scale, covering topics like mood, suicide intentions, sleep problems, weight loss, and feelings of guilt during the past week. For example, feelings of guilt are scored as absent (0), self‐reproach (1), rumination over past errors (2), delusions of guilt (3), or hearing accusatory voices (4). A review of the psychometric properties of the scale found its internal reliability to be adequate (Cronbach's *α* = 0.46–0.97), interrater reliability sufficient (Pearson's *r* = 0.82–0.98), and convergent and discriminant validity to be adequate (Bagby et al. 2004). The HAM‐D was assessed by trained research assistants, with a Type A intraclass correlation coefficient of 0.97 using an absolute‐agreement definition across multiple assessors (Driessen et al. 2013). Patients were included in the RCT if they received a score of 14 or higher, which served as the cutoff for moderate depression. Patients were considered severely depressed when the score was 25 or higher (Ruhé et al. 2005). In the current sample, the HAM‐D scores ranged from 14 to 40 at baseline, 0–34 at posttreatment, and 0–35 at follow‐up.

#### 3.3.2 NEO‐FFI

The NEO‐FFI is a self‐report questionnaire examining personality traits (McCrae and Costa 2004). This shortened version of the NEO‐PI consists of 60 items across five subscales with 12 items each: neuroticism, extraversion, openness to experience, agreeableness, and conscientiousness. Item examples include “I am not a worrier” and “I like to have a lot of people around me.” All items are scored on a 5‐point Likert scale ranging from 0 to 4. The current study will focus on the neuroticism and extraversion subscales (both with a score range of 0–48), with internal consistencies of 0.85 and 0.80, respectively (Sherry et al. 2007). Based on the meta‐analytic review by Samuel and Widiger (2008), a variable constituting avoidant personality traits was calculated by subtracting each participant's score on the extraversion scale (E) from their score on the neuroticism scale (N). As such, we constructed a continuous variable representing avoidant personality traits (low E and high N). The range of this computed variable is −48 to 48, with lower scores (below 0) indicating a lower amount of avoidant personality traits. In the current sample, the computed NEO‐FFI avoidance scores (NEO‐FFI‐AV) ranged from −31 to 42 at baseline, −39 to 39 at posttreatment, and −36 to 36 at follow‐up.

#### 3.3.3 Utrecht Coping List (UCL)

The UCL is a Dutch self‐report questionnaire with 47 items assessing seven dimensions of coping: active confrontation, avoidance, depression, seeking social support, palliative reaction, expressing emotion, and comforting cognitions (Schreurs et al. 1988). According to the psychometric evaluation of the UCL by Sanderman and Ormel (1992), the questionnaire has good factorial and construct validity. All subscales have been found to have satisfactory reliability and stability. The current study will focus on the UCL avoidance subscale (UCL‐AV) to measure avoidant coping. This subscale consists of eight items, which for instance ask about how often one avoids situations that are difficult or how often one waits to see how a situation turns out before getting involved. The items are scored on a 4‐point Likert scale from “*seldom or never*” (1) to “*very often*” (4). The scale ranges from 8 to 32, with an internal consistency of 0.74. In the current sample, the UCL‐AV scores ranged from 8 to 32 at baseline, 9–30 at posttreatment, and 8–28 at follow‐up.

### 3.4 Procedure

Participants were referred by their general practitioner to one of three outpatient mental health clinics in Amsterdam, the Netherlands, and screened for study participation by their intake clinician. Participants meeting eligibility criteria were randomly assigned to either CBT or STPP. Both CBT and STPP consisted of 16 individual sessions, which took place within a 22‐week period. CBT was conducted according to a session‐by‐session treatment protocol with homework assignments for depression (Molenaar et al. 2009) in which behavioral activation and cognitive restructuring were utilized. In the psychodynamic approach (short‐term psychodynamic supportive psychotherapy; de Jonghe 2005), supportive and insight‐increasing techniques were used, with a focus on intrapersonal patterns as well as past and current relationships in order to explore the emotional background of the individual's depressive symptoms.

HAM‐D assessments took place at Week 0 (pretreatment), Week 5, Week 10, Week 22 (posttreatment), and Week 52 (follow‐up). The clinics' ROM, including NEO‐FFI and UCL, took place at Week 0, Week 22, and Week 52. Only data from pretreatment, posttreatment (Week 22), and follow‐up (Week 52) were analyzed.

### 3.5 Statistical Analyses

Multilevel regression analyses were performed with MLwiN 2.35. A significance level of *α* = 0.05 was applied. No outliers or influential cases (Cook's distance) were found, and data were normally distributed for all analyses. Missing outcome data were accounted for by including a time main effect in the multilevel models, allowing participants with baseline scores, but missing posttreatment and follow‐up scores to remain included in the analyses. We followed recommendations by Twisk et al. (2018) to adequately account for baseline differences in outcome measures.

First, to examine the potential moderating effect of baseline avoidance, we estimated a model with HAM‐D as the dependent variable, including main effects for time and the moderator, time × treatment and time × moderator interactions, and a time × treatment × moderator three‐way interaction. We estimated separate models for baseline avoidant personality (NEO‐FFI‐AV) and baseline avoidant coping (UCL‐AV). A significant three‐way interaction was taken to indicate a moderation effect (the relative treatment effects of CBT vs. STPP differing as a function of avoidance).

Second, to determine whether CBT and STPP resulted in significant change of avoidant personality traits (NEO‐FFI‐AV) or coping style (UCL‐AV), we estimated two separate models for each of these independent variables including a time main effect and a time × treatment interaction (Twisk et al. 2018; equation 2c). In these analyses, a significant time main effect indicates a significant change in avoidance, whereas a significant time × treatment interaction indicates a difference between treatment conditions in this change. Attrition was taken into account by the multilevel modeling method.

## 4 Results

A total of 341 individuals took part in the study and were randomly assigned to CBT (*n* = 164) and STPP (*n* = 177). A total of 76 individuals (22%) did not complete ROM questionnaires during the study and therefore could not be included in the analyses, resulting in a total sample size of 265. Before the start of treatment (Week 0), no statistically significant differences were found between the two treatment conditions with regard to depressive symptom severity (HAM‐D; Table 1), avoidant personality traits (NEO‐FFI‐AV), and avoidant coping (UCL‐AV). Observed NEO‐FFI‐AV and UCL‐AV scores at the various assessment moments are listed in Table 2.

**TABLE 1:** Patient characteristics at baseline.

	Total (*n* = 265)		CBT (*n* = 123)		STPP (*n* = 142)		
	*N*	%	*N*	%	*N*	%	
Gender							*Χ* ^2^(1) = 0.308, *p* = 0.579
Male	73	27.5	32	26.0	41	28.9	
Female	191	72.1	91	74.0	100	70.4	
Cultural background							*Χ* ^2^(7) = 5.931, *p* = 0.548
Northwest Europe	158	59.6	77	62.6	81	57.0	
Caribbean	34	12.8	11	8.9	23	16.2	
Marocco	31	11.7	17	13.8	14	9.9	
Turkey	16	6.0	5	4.1	11	7.7	
Other	26	9.8	13	10.6	13	9.2	
Marital status							*Χ* ^2^(4) = 3.219, *p* = 0.522
Married	61	23.0	34	27.6	27	19.0	
Divorced	53	20.0	22	17.9	31	21.8	
Widowed	8	3.0	3	2.4	5	3.5	
Never married	140	52.8	63	51.2	77	54.2	
Other	3	1.1	1	0.8	2	1.4	
Job status							*Χ* ^2^(5) = 5.657, *p* = 0.341
Currently working	105	39.6	47	38.2	58	40.8	
Sickness benefits	45	17.0	27	22.0	18	12.7	
Social security benefits	46	17.4	19	15.4	27	19.0	
Disability benefits	28	10.6	11	8.9	17	12.0	
Student	12	4.5	4	3.3	8	5.6	
Other	27	10.2	14	11.4	13	9.2	
Education level							*Χ* ^2^(7) = 8.019, *p* = 0.331
Low	48	18.2	24	19.5	24	16.9	
Intermediate	127	48.1	54	43.9	73	51.4	
High	83	31.3	44	35.8	39	27.5	
Other	6	2.3	1	0.8	5	3.5	
Previous depressive episodes							*Χ* ^2^(2) = 1.700, *p* = 0.427
None	72	27.2	38	30.9	34	23.9	
1	54	20.4	23	18.7	31	21.8	
2 or more	135	50.9	60	48.8	75	52.8	
	**Mean**	**SD**	**Mean**	**SD**	**Mean**	**SD**	
Age (years)	39.25	10.61	37.84	10.74	40.46	10.38	*t*(263) = 2.022, *p* = 0.044
Depression (HAM‐D)	22.82	5.32	22.88	5.26	22.77	5.40	*t*(263) = −0.157, *p* = 0.875

Abbreviations: CBT, cognitive–behavioral therapy; HAM‐D, Hamilton Depression Rating Scale; STPP, short‐term psychodynamic psychotherapy.

**TABLE 2:** Avoidant personality trait and avoidant coping scores.

	CBT				STPP			
	Mean	SD	*N*		Mean	SD	*N*	
NEO‐FFI‐AV								
Baseline (Week 0)	12.62	11.43	97		13.23	12.38	113	*t*(208) = −0.370, *p* = 0.712
Posttreatment (Week 22)	4.00	13.28	60		5.99	15.89	71	*t*(129) = −0.768, *p* = 0.444
Follow‐up (Week 52)	−0.44	17.18	41		1.61	14.57	28	*t*(67) = −0.516, *p* = 0.608
UCL‐AV								
Baseline (Week 0)	18.21	4.32	111		18.10	4.53	126	*t* (235) = 0.180, *p* = 0.857
Posttreatment (Week 22)	17.67	4.06	64		17.82	4.36	78	*t*(140) = −0.208, *p* = 0.835
Follow‐up (Week 52)	17.52	3.83	46		17.83	3.71	30	*t*(74) = −0.351, *p* = 0.727

Abbreviations: CBT, cognitive–behavioral therapy; NEO‐FFI‐AV, NEO Five Factor Inventory Avoidance Score (range, −48 to 48); STPP, short‐term psychodynamic psychotherapy; UCL‐AV, Utrecht Coping List Avoidance Subscale score (range, 8–32).

Participants in our study were predominantly female (70.1%) with a mean age of 38.91 years (range, 22–64 years). Most participants had a Northwest European background (55.6%) and were educated at an intermediate to high level (77.6%). About half of the patients had two or more previous depressive episodes (48.0%), and one third were experiencing their first depression (31.1%).

Participants with missing ROM data had higher baseline HAM‐D scores (*t*(339) = −3.788, *p* < 0.001), had fewer previous depressive episodes (*Χ* ^2^(2) = 8.455, *p* = 0.014), were more often on social security benefits (*Χ* ^2^(5) = 16.781, *p* = 0.005), and were more likely to report a non‐European cultural background (*Χ* ^2^(3) = 12.252, *p* = 0.007). No significant differences between the two groups were found with regard to age, gender, marital status, and treatment condition (Table S1).

Outcomes of the moderation analyses are provided in Table 3. In contrast with our expectations, avoidant personality traits at baseline were not found to be significantly related to the comparative efficacy of CBT and STPP in terms of depression severity at posttreatment (*β* = −0.001, SE = 0.085, *p* = 0.99) and follow‐up (*β* = 0.028, SE = 0.099, *p* = 0.78). Neither was avoidant coping at baseline related to comparative treatment effects at posttreatment (*β* = 0.045, SE = 0.222, *p* = 0.84) and at follow‐up (*β* = 0.064, SE = 0.251, *p* = 0.80). Avoidant personality traits at baseline were also not associated with treatment outcome in general (irrespective of treatment approach; *β* = 0.64, SE = 0.068, *p* = 0.38), and neither was baseline avoidant coping (*β* = 0.38, SE = 0.179, *p* = 0.83). Depression severity at baseline was, however, positively correlated with avoidant personality traits, *r*(208) = 0.23, *p* < 0.001, though not with avoidant coping, *r*(235) = 0.05, *p* = 0.413.

**TABLE 3:** Moderating effects of baseline avoidant personality and coping on CBT vs. STPP outcome.

	Posttreatment (22 weeks)			Follow‐up (52 weeks)		
	*β*	SE	*p*	*β*	SE	*p*
Avoidant personality						
NEO‐FFI‐AV	0.098	0.038	0.01	0.098	0.041	0.02
Time	−8.486	0.775	< 0.001	−10.866	0.885	< 0.001
Time × Treatment	0.146	0.974	0.85	2.161	1.154	0.06
Time × NEO‐FFI‐AV	0.064	0.068	0.38	0.045	0.080	0.57
Time × Treatment × NEO‐FFI‐AV	−0.001	0.085	0.99	0.028	0.099	0.78
Avoidant coping						
UCL‐AV	0.063	0.101	0.53	0.063	0.108	0.56
Time	−8.782	0.752	< 0.001	−11.075	0.843	< 0.001
Time × Treatment	0.582	0.953	0.54	2.038	1.103	0.06
Time × UCL‐AV	0.038	0.179	0.83	0.118	0.196	0.55
Time × Treatment × UCL‐AV	0.045	0.222	0.84	0.064	0.251	0.80

*Note:* Dependent variable: Hamilton Depression Rating Scale (HAM‐D). Reference category: CBT.

Abbreviations: CBT, cognitive–behavioral therapy; NEO‐FFI‐AV, NEO Five Factor Inventory Avoidance Score; STPP, short‐term psychodynamic psychotherapy; UCL‐AV, Utrecht Coping List Avoidance Subscale.

Outcomes of the analyses assessing change in avoidant personality traits and avoidant coping are presented in Table 4. A significant reduction in avoidant personality traits from baseline to posttreatment (*β* = −7.932, SE = 1.492, *p* < 0.001) and from baseline to follow‐up (*β* = −12.967, SE = 1.789, *p* < 0.001) was observed, with no significant differences between the treatment conditions (*β* = 0.956, SE = 1.948, *p* = 0.62), partially supporting our hypothesis. In contrast and not in line with our hypothesis, no significant change in avoidant coping was observed (*β* = −0.247, SE = 0.527, *p* = 0.60), with no significant difference between treatment conditions at posttreatment (*β* = −0.122, SE = 0.672, *p* = 0.86) nor follow‐up (*β* = 0.806, SE = 0.854, *p* = 0.35).

**TABLE 4:** Change in avoidant personality and coping during CBT and STPP for depression.

	Posttreatment (22 weeks)			Follow‐up (52 weeks)		
	*β*	SE	*p*	*β*	SE	*p*
NEO‐FFI‐AV						
Time	−7.932	1.492	< 0.001	−12.967	1.789	< 0.001
Time × Treatment	0.956	1.948	0.62	2.860	2.691	0.29
UCL‐AV						
Time	−0.247	0.527	0.60	−0.751	0.559	0.18
Time × Treatment	−0.122	0.672	0.86	0.806	0.854	0.35

*Note:* Reference category: CBT.

Abbreviations: CBT, cognitive–behavioral therapy; NEO‐FFI‐AV, NEO Five Factor Inventory Avoidance Score; STPP, short‐term psychodynamic psychotherapy; UCL‐AV, Utrecht Coping List Avoidance Subscale.

## 5 Discussion

The current study examined whether avoidant personality traits and avoidant coping were related to differential treatment outcomes of CBT and STPP for depression. Furthermore, we explored whether depression‐focused CBT and STPP could lead to a change in avoidant personality traits or avoidant coping in depressed adults. We aimed to overcome a common limitation in personality pathology literature by using dimensional rather than categorical measures of avoidance.

Neither baseline avoidant personality traits nor avoidant coping was associated with the differential efficacy of CBT and STPP for depression. These results contradict studies finding structured depression treatment (i.e., CBT) to be most effective for patients with avoidant personality (Barber and Muenz 1996) or any type of comorbid PD (Joyce et al. 2007). These results contradict studies finding structured depression treatment (i.e., CBT) to be most effective for patients with avoidant personality (Barber and Muenz 1996) or any type of comorbid PD (Joyce et al. 2007), depressed patients with anxious and avoidant attachment (McBride et al. 2006), and depression with comorbid anxiety disorders and anxiety symptoms (van Bronswijk et al. 2018). A possible explanation is that these studies compared CBT with IPT instead of the short‐term psychodynamic approach that constituted the comparison in this study. Although IPT focuses on resolving interpersonal problems in the present, STPP approaches psychological issues from a perspective of how we relate and connect with others in the present, and additionally explores challenges and relationships in the past (Markowitz et al. 1998). The fact that CBT was not found to be more effective than STPP in the current study might suggest that STPP's explicit focus on past events is of added value for depressed patients with avoidant personality traits. The active role of the therapist, as well as the explicit focus on resistance in STPP, in order to reduce avoidance and learning to tolerate negative emotions (Leichsenring et al. 2004; McCullough 1999) might have been of further value to prevent avoidant traits from negatively affecting treatment outcome. Future studies comparing all three treatment types (i.e., including head‐to‐head comparisons of CBT, IPT, and STPP) are needed to test these hypotheses and explore the topic in more detail.

We did not find higher levels of avoidance rates associated with poorer treatment outcome in general, contrary to what might be expected based on the meta‐analytic findings of Newton‐Howes et al. (2006, 2014). Mulder (2002) argues that personality pathology does not necessarily worsen depression treatment outcomes, but that findings appear to be dependent on study design. Generally, in depression research, PD are consistently seen as strong predictors for long‐term depressive symptoms (Mulder 2002). One of the major differences between previous research and the current study is the fact that the latter assessed personality with dimensional measures, whereas previous studies like Newton‐Howes et al. (2006, 2014) assessed personality categorically and excluded patients with subthreshold personality problems (i.e., unspecified and other specified PD). These differences in inclusion criteria can have major implications for study outcome, and using categorical data can lead to samples that are less representative of the general clinical population (Samuel and Widiger 2008). The current sample included patients above as well as below the DSM‐5 threshold for avoidant PD diagnosis, being representative of the regular clinical population, and we can conclude that patients benefit from both standard depression treatments, regardless of avoidant personality traits and coping.

Neither CBT nor STPP was superior in reducing avoidant personality traits or coping style. Our findings add to the discussion in the field (Emmelkamp et al. 2006; Svartberg et al. 2004), strengthening the conclusion that no treatment type appears to be clearly superior in treating avoidant personality symptomatology. Avoidant personality features decreased as a result of both depression treatments, whereas avoidant coping appeared to remain unchanged. This is a counterintuitive finding, given that personality traits are generally considered to be more stable than coping style, and the latter has been found to change towards more adaptive coping through treatment (Franken et al. 2001). However, this coping change was observed regarding palliative coping (i.e., distraction or substance use to attenuate emotion), passivity, and socialization, though not necessarily for avoidant coping, which was the focus in this study. The absence of a correlation between avoidant coping and depression is in line with previous findings (Franken et al. 2001) and might indicate that the two are relatively independent constructs. It is also possible that higher avoidant personality scores are a short‐term consequence of depressive symptoms, and not a reflection of a stable trait. Depressive symptoms were, however, controlled for in the statistical model. The observed reductions of avoidant personality traits of 7.9–13.0 points at follow‐up can be considered clinically relevant (Costa et al. 2005). This indicates that psychotherapy aimed at depression can result in an improvement of avoidant personality pathology, which is in line with prior research showing reductions of avoidant personality (Svartberg et al. 2004; Mulder et al. 2010) and changes of NEO‐FFI neuroticism and extraversion after psychotherapy (Costa et al. 2005; Tang et al. 2009).

### 5.1 Strengths and Limitations

A major strength of the current study is the dimensional assessment of personality. In addition, we included a relatively large number of participants and a representative sample of adults with depression and avoidant personality traits commonly seen in clinical practice. Based on these premises (dimensional measures and clinical representativeness), we aimed to replicate previous research findings, attempting to provide results readily applicable to clinical practice.

The following limitations of this study should be addressed in future research. First, we assessed avoidant personality and coping with two specific instruments, and replication with other dimensional measures is needed. As the NEO‐FFI broadly operationalizes subclinical personality dimensions, the pathological variations of avoidance may have been overlooked in this study. In order to measure more specific and maladaptive manifestations of personality traits, the personality inventory of the DSM‐5 (PID‐5; Krueger et al. 2012) offers a viable alternative for future studies (Pocnet et al. 2018). To the best of our knowledge, the UCL's responsiveness has never been investigated, and it is possible that this instrument's avoidance subscale was not sensitive enough to detect a change in coping style. Beutler et al. (2011) introduced an internalization ratio formula for MMPI‐2 scores that might be more responsive to determine coping style change. Alternatively, the Cognitive–Behavioral Avoidance Scale (CBAS; Ottenbreit and Dobson 2004) might be a suitable instrument to determine changes in avoidant coping in future research. Second, results might have been affected by the fact that participants with missing baseline ROM data, which could not be included in the analysis, were more depressed at baseline, had fewer depressive episodes in the past, and were more likely to be of non‐European origin. Hence, it remains uncertain whether avoidance reduces as much for patients with high depression at baseline, patients experiencing their first depressive episode, and patients with non‐European ethnicity. Related, although the multilevel modeling method applied can account for missing data, the second aim of the study is limited by the relatively high attrition rates.

### 5.2 Clinical Implications and Suggestions for Future Research

The results of this study support the idea that widely used depression treatments like CBT or STPP are valuable treatment options for patients with avoidant personality. Both treatments can be offered to patients with depression, regardless of avoidant personality traits or coping, and both treatments can reduce depressive symptoms as well as avoidant personality traits. As such, the choice between CBT and STPP may be based on patient preference, which might improve patient motivation, adherence, and long‐term treatment effects (Cuijpers et al. 2011). As mentioned above, future RCTs comparing CBT, IPT, and STPP are needed to test the hypothesis that patients with avoidant personality benefit more from CBT and STPP than from IPT. In addition, comparisons with other therapies would help to establish whether these findings generalize to other interventions (e.g., schema‐focused therapy and acceptance and commitment therapy).

The lower amount of avoidant personality traits at the end of treatment can potentially improve long‐term prognosis and outcome of further treatment, as less avoidance can facilitate behavioral activation, which is an important element within recovery from depression (Gunthert et al. 2005).

We recommend further research using alternative dimensional measures of personality and coping to replicate the current findings. In addition to avoidance, future studies might focus on concepts like dependency, trustfulness, or perfectionism (Blatt 2004; Smith et al. 2021), as these may have an impact on the therapeutic relationship or adherence to topics discussed in therapy sessions. The use of dimensional measures remains a relevant and interesting aspect for several reasons previously discussed. Regarding the current shift from categorical to dimensional views of PD, our results align with the transition between these eras. The dimensional approach to avoidance can be applied to patients who score below or above the threshold for avoidant PD. This makes our results more readily applicable to clinical practice.

## Funding

Data collection for this study was supported by Wyeth Pharmaceuticals, the Netherlands; Arkin Mental Health Care, Amsterdam; Pro Persona Mental Health Care; and the Faculty of Psychology and Education, Department of Clinical Psychology, VU University, Amsterdam. Dr. Driessen's contributions to this research were supported by a grant from the Dutch Research Council (016.Veni.195.2156806).

## Conflicts of Interest

Dr. Van is the medical director of the NPI and chief editor of the Dutch Journal of Psychiatry. Mr. Don receives royalties from Springer Media. Dr. Driessen has received grants from the American Psychoanalytic Association, the Dutch Psychoanalytic Funds, and the Dutch Research Council. All other authors report no financial relationships with commercial interests.

## Supporting information


**Table S1:** Comparison of participants with and without completed ROM.

## Data Availability

The data that support the findings of this study are not publicly available due to their sensitive nature. However, data requests, including a detailed plan for how they will be used, can be directed to the research group for consideration via email to the corresponding author (ellen.driessen@ru.nl). Access can be obtained after approval of a study proposal that gives rise to a signed data access agreement.
