# Supplementary material for: Avoidant Personality Traits and Avoidant Coping in Cognitive–Behavioral Therapy vs. Short‐Term Psychodynamic Psychotherapy for Adult Depression
Source: Personal Ment Health. 2026 May 3;20:e70081. doi: 10.1002/pmh.70081 (PMC13135863; doi:10.1002/pmh.70081)
Supplement: Supplementary file 1 — Table S1: Comparison of participants with and without completed ROM. [file PMH-20-0-s001.docx]

Supplemental Table A – Comparison of participants with and without completed ROM

|  | Total (n=341) | | ROM (n=265) | | Missing ROM (n=76) | |  |
| --- | --- | --- | --- | --- | --- | --- | --- |
|  | N | % | N | % | N | % |  |
| Gender |  |  |  |  |  |  |  |
| Male | 102 | 29.9 | 73 | 21.5 | 29 | 8.5 | ꭕ²(1)=3.102, *p* = .089 |
| Female | 239 | 70.1 | 191 | 56.2 | 47 | 13.8 |  |
| Treatment Condition |  |  |  |  |  |  |  |
| CBT | 164 | 48.1 | 123 | 36.1 | 41 | 12.0 | ꭕ²(1)=1.342, *p* = .298 |
| STPP | 177 | 51.9 | 142 | 41.6 | 35 | 10.3 |  |
| Cultural Background |  |  |  |  |  |  |  |
| Northwest Europe | 188 | 55.6 | 160 | 47.3 | 28 | 8.3 | ꭕ²(3)=12.252, *p* = .007 |
| Caribbean | 46 | 13.6 | 34 | 10.1 | 12 | 3.6 |  |
| Northern Africa | 75 | 22.2 | 50 | 14.8 | 25 | 7.4 |  |
| Other | 29 | 8.6 | 21 | 6.2 | 8 | 2.4 |  |
| Marital Status |  |  |  |  |  |  |  |
| Married | 80 | 23.7 | 61 | 18.0 | 19 | 5.6 | ꭕ²(4)=1.299, *p* = .886 |
| Divorced | 69 | 20.4 | 53 | 15.7 | 16 | 4.7 |  |
| Widowed | 10 | 3.0 | 8 | 2.4 | 2 | 0.6 |  |
| Never Married | 176 | 52.1 | 140 | 41.4 | 36 | 10.7 |  |
| Other | 3 | 0.9 | 3 | 0.9 | 0 | 0.0 |  |
| Job Status |  |  |  |  |  |  |  |
| Currently working | 130 | 38.8 | 105 | 31.3 | 25 | 7.5 | ꭕ²(5)=16.781, *p* = .005 |
| Sickness benefits | 55 | 16.4 | 45 | 13.4 | 10 | 3.0 |  |
| Social security benefits | 74 | 22.1 | 46 | 13.6 | 28 | 8.4 |  |
| Disability benefits | 32 | 9.6 | 28 | 8.4 | 4 | 1.2 |  |
| Student | 14 | 4.2 | 12 | 3.6 | 2 | 0.6 |  |
| Other | 30 | 9.0 | 27 | 8.1 | 3 | 0.9 |  |
| Education Level |  |  |  |  |  |  |  |
| Low | 67 | 20.0 | 48 | 14.3 | 19 | 5.7 | ꭕ²(7)=6.485, *p* = .471 |
| Intermediate | 159 | 47.5 | 127 | 37.9 | 32 | 9.6 |  |
| High | 101 | 30.1 | 83 | 24.8 | 18 | 5.4 |  |
| Other | 8 | 9.0 | 6 | 1.8 | 2 | 0.6 |  |
| Previous depr. episodes |  |  |  |  |  |  |  |
| None | 103 | 31.1 | 72 | 21.8 | 31 | 9.4 | ꭕ²(2)=8.455, *p* = .014 |
| 1 | 69 | 20.8 | 54 | 16.3 | 15 | 4.5 |  |
| 2 or more | 159 | 48.0 | 135 | 40.8 | 24 | 7.3 |  |
|  | Mean | SD | Mean | SD | Mean | SD |  |
| Age (years) | 38.91 | 10.30 | 39.25 | 9.08 | 39.25 | 10.61 | t(339)=-1.136, *p* = .257 |
| Depression (HAM-D) | 23.40 | 5.35 | 22.82 | 5.33 | 25.40 | 4.96 | t(339)=3.788, *p* = .000 |

Note. HAM-D = Hamilton Depression Rating Scale, ROM = Routine Outcome Monitoring
